# An effective method for small objects detection based on MDFFAM and LKSPP

**DOI:** 10.1038/s41598-024-60745-9

**Published:** 2024-05-03

**Authors:** Zhoutian Xu, Yadong Xu, Manyi Wang

**Affiliations:** School of Mechanical Engineering, Nanjing University of Science and Technology, Nanjing, 210094 China

**Keywords:** Small objects detection, Large kernel, Attention mechanism, Subtle faults of machine, Computer science, Mechanical engineering

## Abstract

Object detection is one of the research hotspots in computer vision. However, most existing object detectors struggle with the identification of small targets. Therefore, the paper proposes two modules: the MDFFAM (Multi-Directional Feature Fusion Attention Mechanism) and the LKSPP (Large Kernel Spatial Pyramid Pooling), to enhance the detector's effectiveness in identifying subtle faults on the surface of mechanical equipment. LKSPP aims to expand the receptive field to capture high-level semantic features through large kernels. Meanwhile, the MDFFAM allows the network to efficiently utilize spatial location information and adaptively recognize detection priorities. In the detection task, MDFFAM effectively captures feature information in three spatial directions: width, height, and channel, with the location information fully utilized to establish stable long-range dependencies. Moreover, LKSPP boasts a larger receptive field and imposes less computational burden compared to the SPPCSPC by YOLOv7. Finally, experiments demonstrate that the proposed module effectively improves the detection accuracy for small targets, surpassing the state-of-the-art object detector, YOLOv7. Remarkably, MDFFAM incurs almost negligible computational overhead.

## Introduction

Nowadays, fault diagnosis systems are indispensable components of modern industry. Thanks to the development of AI, intelligent monitoring and real-time decision-making are poised to be major features of future fault diagnosis systems. While most existing mechanical fault diagnoses are based on sensor signals, uncertainties such as complex operating conditions, huge diagnostic systems, and human errors in data processing make it difficult to make real-time decisions through sensor-transmitted fault information. Object detection technology for monitoring key mechanical components can not only reduce the reliance on sensors and simplify the structure of the diagnostic system but also greatly improve the monitoring efficiency and mitigate potential issues caused by human errors.

Defect detection differs from tasks like pedestrian detection and face recognition tasks. Firstly, the difference lies in the feature size; defects on mechanical surfaces are generally subtle and complex, such as fine cracks and densely distributed pitting. Even with a sufficient sample number, it is difficult for the network to fully learn the local information of defects. Secondly, the high similarity between defect types, such as pitting and corrosion, can easily trigger false alarms from the detector. Therefore, it remains an extremely challenging task to improve the recognition accuracy of defect detection.

The mainstream real-time object detectors are divided into two classes, namely the YOLO series^1–6^ and the FCOS^7,8^ series. It is well known that the detection effectiveness of FCOS decreases dramatically when the number of detected targets increases and the size is small. From the success of YOLOv3^5^, YOLOv4^2^, and now YOLOv7^9^, the YOLO series real-time object detector has become the benchmark detection algorithm for computer vision tasks. Nevertheless, the overall framework of most detectors uses small kernels. Although the network can be deepened by continuously stacking the number of layers, this will undoubtedly increase the complexity of the network, and the detection area due to the small receptive field is too small, which can easily trigger global information loss and reduce the recognition rate of the detector. Current researches on small object detection focus on feature fusion^10,11^ and feature enhancement^12^, ignoring the essential location information. Most fusion methods simply connect features at different stages, which tend to add redundant information and fail to establish solid long-range dependencies. Other vision-based approaches are exposed to the following issues: simple classification of defects^13–15^, insufficient use of the positional information in the features^16,17^ and inadequate attention to high-level semantic features. As a consequence, in the context of the great fire of attention mechanism, the paper explores the inherent potential of large kernels and tries to incorporate the attention mechanism in the detector structure, hoping to improve the performance of the detector for small objects by integrating the advantages of both.

The impact of ViTs^18^ (Vision Transformers), which borrows transformer^19^ from the NLP (Natural Language Processing) domain, on computer vision tasks has been tremendous, and many scholars believe that this is mainly attributed to the self-attention mechanism in transformer^20–23^. In vision tasks, MHSA (Multi-Head Self-Attention) divides the input images into multiple patches and takes parallel computation, meaning the data has a global receptive field for each layer after its processing. The kernels in CNNs^24,25^ (Convolutional Neural Networks) are small, and the convolutional layer with kernel 3 × 3 is used as the main component of the model for its few parameters. At the same time, stacking the small kernels can enhance the nonlinear representation of the model. Despite the common use of small kernels, the small receptive field caused by small kernels makes the detection area of the model too small to obtain rich global information, which also reduces the generalization ability of the model. Ding X^26^ innovatively uses 31 × 31 super-large kernels in traditional convolutional neural networks and achieves 87.8% accuracy on ImageNet. Liu Z^27^ frontloads the large 7 × 7 depthwise conv in the ConvNeXt module to obtain a rich global receptive field. The large window employed by swin transformer^28^ in the attention mechanism can also be seen as a variant of the large receptive field. Han Q^29^ replaces the MHSA in the swin transformer with 7 × 7 depthwise conv and obtains a comparable performance with the original structure. ConvMixer^30^ uses a 9 × 9 conv to replace the mixing step in ViTs and outperformed ViTs in terms of performance.

The simple yet effective paradigm of large kernel design can significantly improve model performance, derived from reconsideration of the structure itself. The exploration of a potential connection between the large kernel and the attention mechanism also presents a promising research direction. It should be noted that the attention mechanism is different from the design paradigm of the large kernel. While the attention mechanism is based on the up-down connection between the input tensors and employs a weighted average operation to dynamically calculate attention weights for each pixel, it facilitates the flexibility of the module to focus on different regions and capture more effective information features. Commonly used attention mechanisms are SENet^31^, CBAM^32^, and CA^33^. Among them, SENet and CBAM employ the attention mechanism as an expansion mechanism of the convolution module. Conversely, SAN^34^ and BoTNet^35^ believe the attention module can replace the traditional convolution. On the other hand, the attention mechanism imposes a substantial computational overhead compared to traditional convolution, often leading to computational bottlenecks. AA-ResNet^36^ and Container^37^ integrate the attention mechanism and convolution into a unified module, but the architecture is not conducive to the design of separate paths for each module. SCNet^38^, NLNet^39^, and GSoP-Net^40^, which utilize non-local self-attention networks to capture different types of spatial features, tend to overlook the resource-intensive computational burden of the self-attention mechanism. Therefore, existing studies mainly treat the attention mechanism as a separate part or expansion module and fail to fully utilize the advantages of both the large kernel and the attention mechanism.

To solve the limitations observed in the aforementioned works, this paper introduces an attention mechanism and a large kernel module in the object detector to enhance the model performance. Large kernels directly augment the effective receptive field while partially avoiding the optimization problem caused by increasing model depth. It is widely recognized that large kernels are susceptible to transition smoothing, and the parameters and computation of large kernels are significantly higher than those of smaller counterparts, which potentially leads to gradient explosion. To maximize the effective use of large kernels, the paper proposes LKSPP (Large Kernel Spatial Pyramid Pooling) and summarizes four design principles: (1) introduce reverse bottlenecks, (2) implement front large kernels, (3) establish serial connections, and (4) emphasize the importance of shortcut. In addition, since large kernels struggle to account for local features, they are coupled with small convolutional layers to enhance the model's capacity to capture features at a local scale.

Furthermore, the paper proposes a new attention mechanism, namely MDFFAM (Multi-Directional Feature Fusion Attention Mechanism). To avoid the loss of location information induced by 2D global pooling, channel attention is decomposed into three spatially oriented feature codes for the efficient convergence of spatial location information into the attention map. Specifically, 3D global pooling layers are employed to break the input into three feature-aware maps with different spatial directions (height, width, and channel), each aggregating the input features in its corresponding direction. The resulting feature maps with location-specific information are then encoded into three attention maps. These maps undergo convolution and pooling along their respective directions to further capture the directional feature information. Each feature map independently captures long-range dependencies within the input feature maps along its corresponding direction.

## MDFFAM (multi-directional feature fusion attention mechanism)

### Multi-directional information embedding

**Figure 1:**
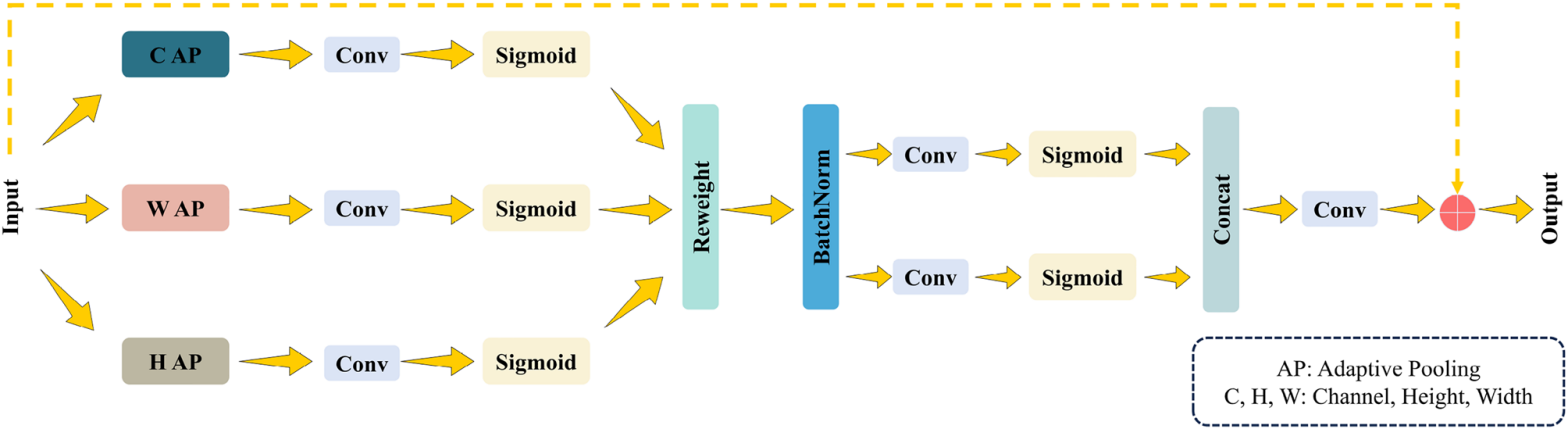
Structure of the MDFFAM.

In the channel attention mechanism, global pooling is commonly used to encode spatial information. However, this approach tends to pass global spatial information into the channel information, making it difficult to consistently provide the positional information necessary to capture the spatial structure. MDFFAM uses precise positional information to encode features along the three spatial directions: channel, height, and width. Assuming the input is denoted as $X\in {\mathbb{R}}^{C\times H\times W}$, the features along the three spatial directions are encoded using three-dimensional adaptive pooling layers with pooling kernels of (C, 1, 1), (1, H, 1), and (1, 1, W), respectively. Specifically, in the width and height directions, where the number of channels is 1, the resulting output can be formulated as (Fig. 1):

1 $${y}_{c=1}^{h}\left(h\right)=\frac{1}{W}\sum_{0\le i<W}{x}_{c=1}\left(h,i\right),$$

2 $${y}_{c=1}^{w}\left(w\right)=\frac{1}{H}\sum_{0\le j<H}{x}_{c=1}\left(j,w\right),$$

where ${y}_{c=1}^{h}\left(h\right)$ is the output at height h, ${y}_{c=1}^{w}\left(w\right)$ is the output at width w. The convolutional layer with a fixed kernel size provides the input X directly; hence, it can be considered a collection of local descriptors. Similarly, the result in the C × 1 × 1 channel direction can be expressed as:

3 $${y}^{c}\left(c\right)=\sum_{0\le k<C}{x}_{c}\left(k\right)$$

The above three formulas enable the decomposition of input X into three feature encodings along different spatial directions, forming a set of spatial direction-sensitive quantities and aggregating feature information along C, H, and W spatial directions. Compared with the SE block that generates individual feature vector, MDFFAM retains precise location information and establishes more robust long-range dependencies.

### Attention generation

In the second step, features are captured along the three spatial directions and generate multi-directional attention. The details are as follows: the three spatial directional features derived from Eqs. (1), (2), and (3) are successively convolved. After applying the Sigmoid activation function, the feature aggregation maps ${g}^{h}$, ${g}^{w}$, and ${g}^{c}$ serve as the attention weights for the different spatial directions, expressed as:

4 $${g}^{h}=\delta \left(Conv\left({y}_{c}^{h}\right)\right),$$

5 $${g}^{w}=\delta \left(Conv\left({y}_{c}^{w}\right)\right),$$

6 $${g}^{c}=\delta \left(Conv\left({y}^{c}\right)\right),$$

where $Conv$() is a convolutional layer with a 1 × 1 kernel and output channel c, $\delta ()$ is the Sigmoid activation function. ${g}^{h}\in {\mathbb{R}}^{C\times H\times 1},{g}^{w}\in {\mathbb{R}}^{C\times 1\times W}$, and ${g}^{c}\in {\mathbb{R}}^{C\times 1\times 1}$ are the attention weights after feature extraction and mapping along the three directions of height, width, and channel. Next, the three attention weights are fused to obtain $f$:

7 $$f={g}^{c}*{g}^{h}*{g}^{w}.$$

After conversion by Eq. (7), the feature attention weight $f\in {\mathbb{R}}^{C\times H\times W}$ for the three directions of fusion is obtained. BatchNorm is subsequently applied to $f$ to prevent the network from overfitting while simplifying the structure. The normalization result is divided into feature maps with the same number of channels by two convolutional layers, i.e., ${f}^{h}\in {\mathbb{R}}^{\frac{C}{r}\times H\times 1}$, ${f}^{w}\in {\mathbb{R}}^{\frac{C}{r}\times 1\times w}$. The parameter r is the reduction ratio used to control the module size. Then, the Sigmoid activation function is applied to each of the two feature maps and the results are concatenated.

8 $${f}^{h}=Conv\left(f\right),$$

9 $${f}^{w}=Conv\left(f\right),$$

10 $$G=Concat\left(\delta \left({f}^{h}\right),\delta \left({f}^{w}\right)\right),$$

${\text{where }} \delta ()$ is the Sigmoid activation function and $G$ is the result after concatenation. A convolution operation on $G$ adjusts the number of channels and adds it to the input *X* to obtain the final output of the entire mechanism:

11 $$Output=X+Conv\left(G\right).$$

MDFFAM distinguishes itself from channel attention by considering the importance of different channels and encoding the information in both high and wide spatial directions. This allows the detector to capture the features along different directions and effectively use the location information to establish solid long-range dependencies that assist the model in object identification.

## LKSPP (large kernel spatial pyramid pooling)

**Figure 2:**
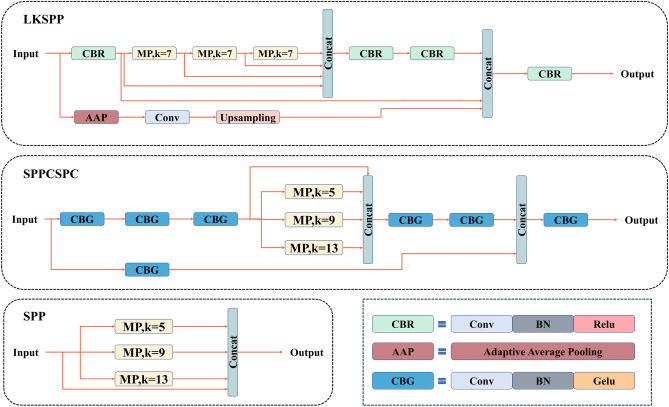
Schematic comparison of the proposed LKSPP with SPCSPC and SPP.

In CNNs, the requirement of fixed input size is usually met by cropping and stretching, which can bring about image distortion and decreased detection accuracy of the model for images. SPP^41^ is an effective solution. Regardless of the input size, the output size after the SPP layer remains fixed, which reduces the risk of overfitting. The feature of multi-size feature fusion enhances network robustness. Figure 2 illustrates three spatial pyramid pooling structures: SPP in Yolov5^42^, SPPCSPC in Yolov7, and LKSPP. SPP, the simplest of the three, uses three max pooling layers to compute the input in parallel. The pooling layers are chosen with large kernels to expand the receptive field. Finally, the original input is stitched with the three pooled results using shortcuts. The SPPCSPC used in Yolov7 follows the same pooling layer design as SPP, with three pooling layers connected in parallel and kernel sizes of 5, 9, and 13. However, before the pooling operation, three convolutional layers are introduced, in which the convolutional kernel of 3 expands the receptive field, making the receptive field obtained by the pooling part of SPPCSPC larger than that of SPP. Moreover, stacking multiple CBG modules effectively increases the depth of the model.

Both SPP and SPPCSPC use large-kernel pooling layers to further illustrate the importance of large receptive fields. However, they have limitations in their structures. SPP simply designs three large kernels in parallel, which increases the computational load in exchange for an extended receptive field and impacts inference speed. SPPCSPC adds many elements to SPP, such as convolutional layers, normalizations, and activation functions, to effectively increase the module depth and reduce the risk of overfitting. The convolutional layer before the pooling operation also helps the module to expand the receptive field. However, SPPCSPC does not take into account the design idea of reverse bottleneck, and the computational burden brought by simply using convolutional layers to expand the receptive field is relatively heavy.

To address the above issues, LKSPP is proposed, with the following design principles: (1) Introduce a reverse bottleneck: the hidden dimension of the module is larger than the input dimension. The design, similar to Transformer’s MLP module and ConvNets, effectively reduces module computation. For instance, ConvNeXt uses reverse bottlenecks and gives the task of changing the channel dimension to 1 × 1 convolutions, which significantly cuts down network FLOPs while enhancing accuracy. In LKSPP, this reverse bottleneck design is reflected in the three convolutional layers after the pooling operation, all employing 1 × 1 kernels. This ensures parameter reduction while expanding channel numbers. All convolutional layers maintain the input feature map’s size and only modify the channel dimension. (2) Implement a front large kernel pooling layer. In the network, pooling layers with large kernels should steer clear of channel number increase calculation. Hence, the reverse bottleneck is positioned at the end of the module while the pooling part is front-loaded. Most of the computational tasks are still handled by 1 × 1 convolutional kernels with output channels halved compared to input channels. This design can further reduce the parameters and computations for the large kernel pooling layer. (3) Establish a serial connection method. Both SPP and SPPCSPC use parallelism to connect large kernel pooling layers. In this way, direct use of large kernels incurs a substantial computational burden, especially for a pooling layer with a 13 × 13 kernel size. In contrast, a serial approach is more reasonable compared to the design paradigm of direct use of multiple large kernels in parallel. SPPF^42^ sequentially connects three pooling layers with 5 × 5 kernels, resulting in a significant speedup with improved performance. LKSPP concatenates three pooling layers with large kernels in serial, each employing the same kernel of 7 × 7. Obviously, the pooling part of the LKSPP boasts the greatest receptive field. 4) Incorporate a global receptive field path. In the design principles for large kernels, shortcuts remain crucial. Similarly, LKSPP introduces a shortcut and adds a global receptive field to this shortcut path. Specifically, input feature maps for each channel are compressed to a 1 × 1 size through an adaptive average pooling layer to facilitate global feature extraction for each channel. Then, a 1 × 1 convolution layer captures information from the extracted global features in a deeper step. Finally, the convolved output restores the feature size of each channel from 1 × 1 to the original size through the Upsampling module. Given the four points, LKSPP experiences a significant reduction in parameters and computations compared to SPPCSPC with a larger receptive field.

## Slim-YOLO

**Figure 3:**
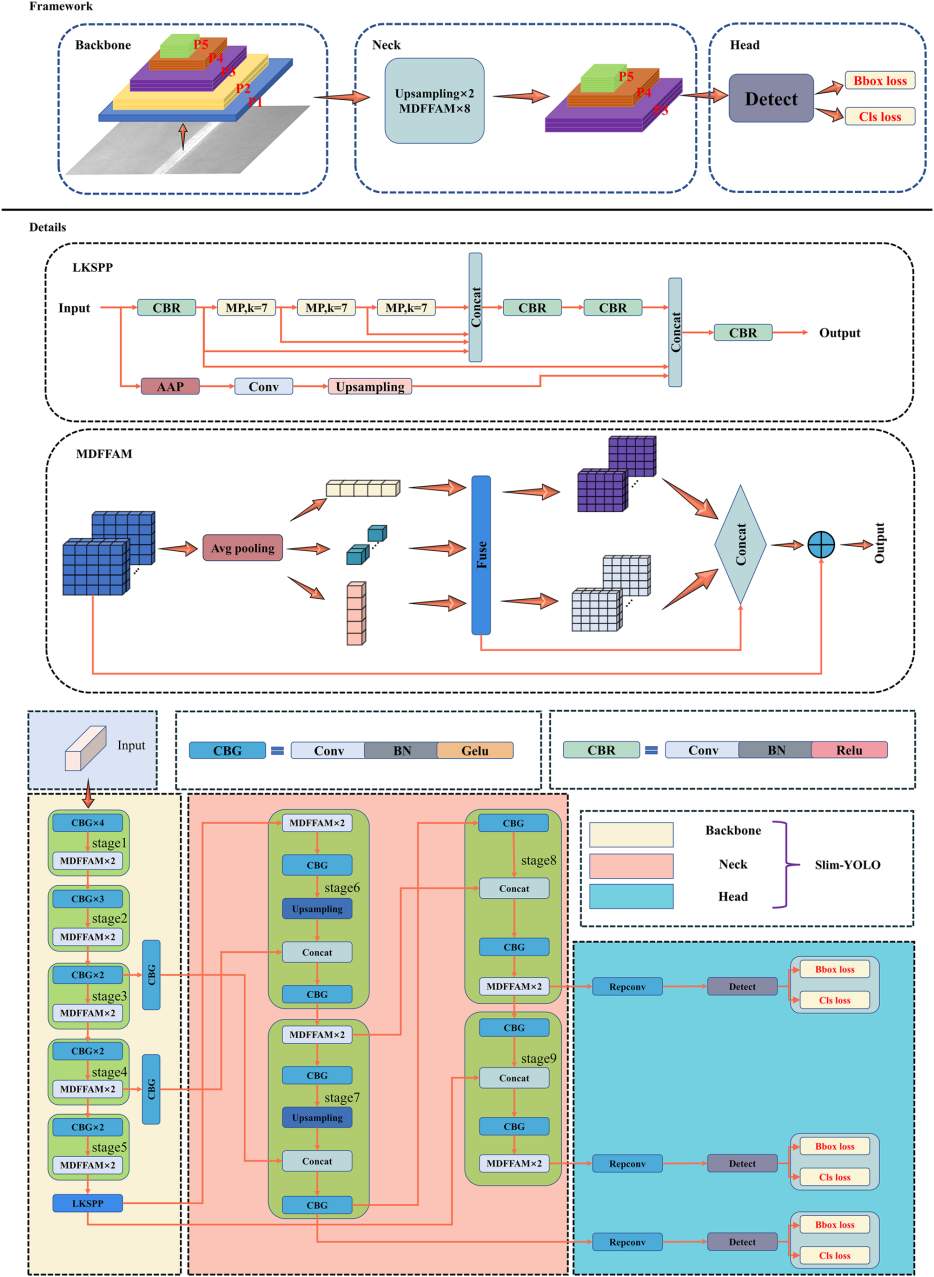
Structure of the Slim-YOLO.

To demonstrate the effectiveness of LKSPP and MDFFAM in improving the performance of the object detector, these two modules serve as the cores in constructing the model, which is referred to as Slim-YOLO. The overall framework of Slim-YOLO is depicted in Fig. 3 and comprises three major components: backbone, neck, and head.

Backbone: The role of the backbone part is mainly to extract features from the input. It is divided into five stages, each generating feature maps with varying sizes and channel dimensions. As the network deepens, the size of the feature map decreases and the channel dimension increases. Specifically, to obtain rich feature information early in the extraction process, several CBG modules are applied at each stage, i.e., Convolution Layer + BatchNorm + Activation Function Gelu. After CBG, two MDFFAM modules are introduced to enhance the utilization of location information. MDFFAM extracts features from the input along three spatial directions and fuses the resulting feature maps, which effectively boosts the robustness of the network. Given that the detector obtains rich local features in the initial part, four CBGs are used in stage 1, gradually decreasing to two in the last three stages. The backbone continues to pass the extracted feature maps to the neck for further feature fusion and reprocessing.

Neck: First, LKSPP performs a pooling operation on the feature maps extracted by the backbone. A serial large kernel pooling layer is designed to filter out redundant features, accurately retain critical information, reduce network parameters, and enhance the fused feature information. Then, two Upsampling modules are utilized to augment the resolution of the feature maps. The feature map (P4) generated in stage 4 is fused with the output feature map of the Upsampling module in stage 6. Similarly, the output feature map of the Upsampling module in stage 7 is fused with the feature map (P3) generated in stage 3. Stage 8 and stage 9 share a similar architecture, where a CBG module with 3 × 3 kernel is added before and after the Concat layer to enhance the ability to capture local features. MDFFAM makes full use of the spatial location information of the CBG-processed feature maps and establishes solid long-range dependencies between the modules.

Head: This part is mainly responsible for the localization and classification of the previously processed feature maps. The processing means usually focus on non-maximal value suppression methods and other versions, such as soft NMS^43^ and weighted NMS^44^. In the head, RepConv is used to expedite model inference during deployment. During training, RepConv consists of three branches: 1 × 1 convolution, 3 × 3 convolution, and BatchNorm layer. During deployment, the model fuses the convolutional layers and BatchNorm layers of the three RepConv branches with a reparameterization technique, equivalently into a VGG-like structure. RepConv is subsequently used behind each of the three feature maps in the final output to further accelerate the inference. Eventually, the detection head calculates the bounding box loss and classification loss for localization.

## Experiment

### Experiment preparation

This paper uses the NEU-DET^45^ surface defect detection dataset, which contains six typical mechanical surface defects, i.e., Rolled-in scale (Rs), Patches (Pa), Crazing (Cr), Pitted surface (Ps), Inclusion (In), and Scratches (Sc). Each defect type comprises 300 images, for a total of 1800 images. The dataset is divided into three subsets: a test set with 1134 images, a validation set with 126 images, and a training set with 540 images.

All experiments are based on the Pytorch environment and are executed from scratch without pre-trained models. In the comparative and ablation experiments, only the module is changed, with the parameter settings consistent with the baseline YOLOv7. All models undergo training for 200 epochs with an input image size of 320 × 320.

The hardware configuration for the experiments includes an Nvidia GeForce RTX3060 graphics card, an AMD Ryzen 7 5800H with a Radeon Graphics processor operating at speeds of up to 3.2 GHz, and 16 GB of RAM.

### Baseline

To verify the superiority of the proposed module, the previous versions of the YOLO series and the most advanced object detector, YOLOR, are selected as baselines. Slim-YOLO is compared with baselines, and the experimental results are shown in Table 1.

**Table 1:** Slim-YOLO vs. baseline.

Models	Param. (M)	FLOPs (G)	mAP50 (%)
Slim-YOLO	34.6	76.8	71.7
YOLOv3	61.5	154.6	70.4
YOLOv3-SPP^5^	62.6	155.5	69.4
YOLOv4-CSP^6^	52.5	119	66.9
YOLOv7	36.5	103.2	71.2
YOLOv5L^42^	46.1	107.7	69.9
YOLOR-CSP-X^46^	96.4	224.9	57.9
YOLOR-CSP^46^	52.5	119.0	60.6
YOLOR-D6^46^	150.9	232.0	29.4
YOLOR-P6^46^	36.8	80.2	24.6
YOLOR-W6^46^	79.3	111.9	58.9
YOLOR-E6^46^	115.1	168.9	38.1

In comparison with the YOLO series, Slim-YOLO exhibits the highest ${mAP}_{50}$, with a 4.8% improvement over the least accuracy YOLOv4-CSP, and even a 0.5% enhancement over YOLOv7, the most advanced real-time object detector currently available. While Slim-YOLO demonstrates an absolute advantage in terms of accuracy, it does impose a slight computational burden on the hardware. First of all, the parameters of Slim-YOLO are only 34.6 M, which is 5.5% less than YOLOv7 and even 80.9% less than YOLOv3-SPP. Furthermore, in terms of computation, although YOLOv7 is undoubtedly the smallest in the YOLO series with only 103.2G, Slim-YOLO places a much smaller computational burden, 34.4% less than YOLOv7, which fully illustrates that Slim-YOLO's core modules, MDFFAM and LKSPP, are lightweight.

Similarly, in comparison with the detectors of the YOLOR series, Slim-YOLO outperforms the top three indicators. In terms of the parameters, it is 6.4% fewer than YOLOR-P6, the lowest in the YOLOR series. In Flops, it is 4.4% less than YOLOR-P6 and even only one-third of YOLOR-CSP-X. Slim-YOLO also demonstrates superior accuracy performance, with an 11.1% improvement over YOLOR-CSP, which has the highest accuracy in the YOLOR series.

How to effectively improve the model accuracy and mitigate the increase in computational burden has been the key to measuring the effectiveness of the module. By comparing with the baselines, it is evident that Slim-YOLO has successfully balanced both accuracy and computational cost, which further demonstrates that the core components of Slim-YOLO, MDFFAM, and LKSPP, markedly enhance model accuracy.

**Figure 4:**
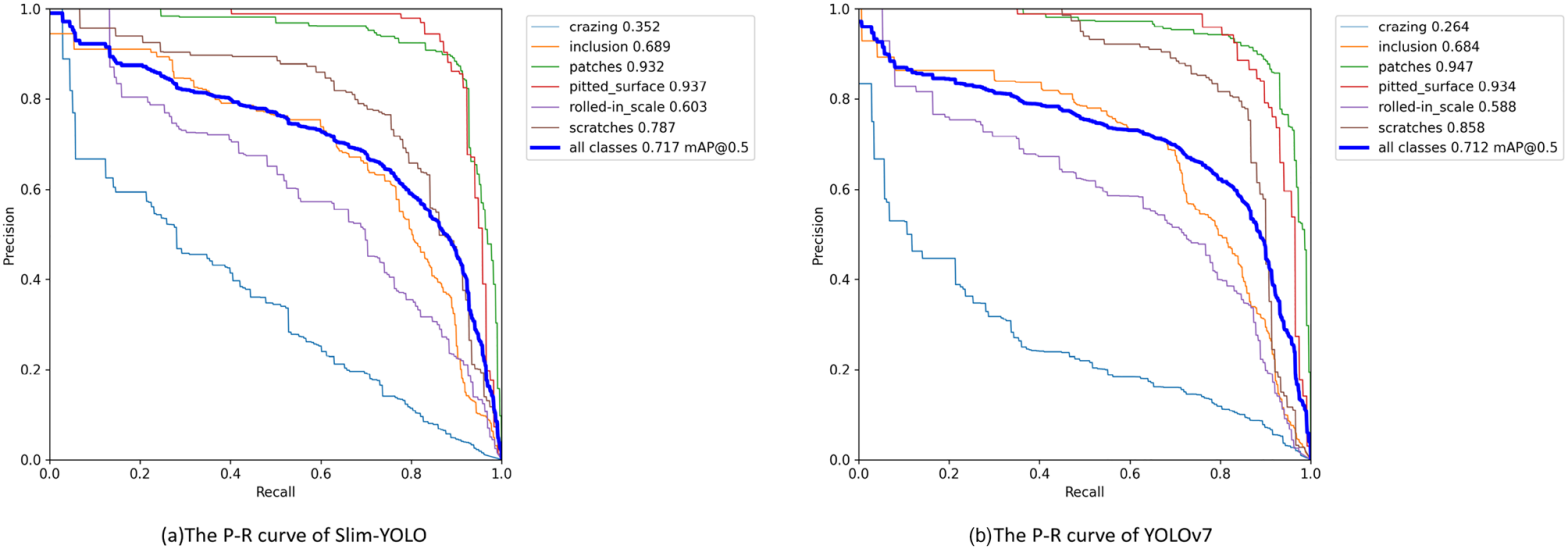
P-R curves of the Slim-YOLO and YOLOv7.

Figure 4 illustrates the P-R curves of YOLOv7, the most advanced of the YOLO series, and the proposed Slim-YOLO. In the category accuracy, Slim-YOLO exceeds YOLOv7 in four categories, with the most significant improvement seen in ‘Crazing’ at 8.8%. It is worth noting that the computational burden of Slim-YOLO is much smaller than that of YOLOv7. Slim-YOLO outperforms YOLOv7 in terms of detection accuracy for all categories, and its computational burden is notably lighter than that of YOLOv7.

**Figure 5:**
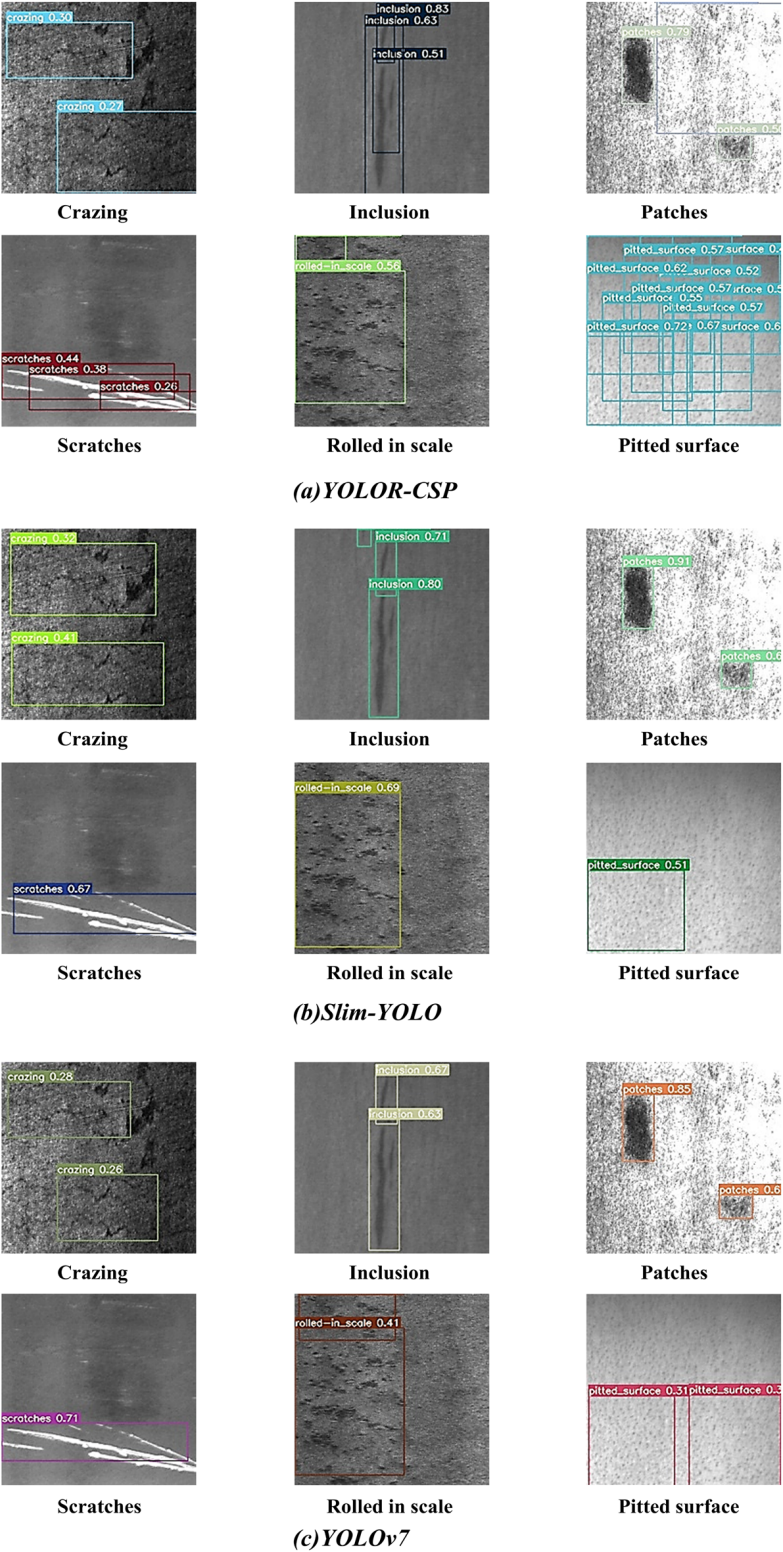
Effectiveness of different detectors in detecting defects.

To visualize the detection performance of Slim-YOLO on defect features, six defect types in the dataset are randomly selected for experiments. YOLOv7 and YOLOR-CSP, the top performers in the YOLO and YOLOR series, function as the baselines, and the results are shown in Fig. 5. The distribution complexity of each defect type varies, with ‘Rolled in scale’ and ‘Crazing’ exhibiting the highest distribution complexity, which leads to a lower detection accuracy for these two types of defects using the baselines. Slim-YOLO achieves the highest detection accuracy in ‘Rolled in scale’, 28% and 13% higher than YOLOv7 and YOLOR-CSP, respectively. It also demonstrates the optimal detection accuracy in ‘Crazing’, a surface defect type highly similar to ‘Inclusion’. In ‘Scratches’, Slim-YOLO displays slightly lower accuracy than YOLOv7, while YOLOR-CSP exhibits the lowest accuracy and overlapping detection frames. In the remaining three defects, Slim-YOLO outperforms the benchmark model and achieves 91% detection accuracy for ‘Patches’. These results demonstrate that Slim-YOLO, with the introduction of MDFFAM, is better equipped to capture the positional information of the features and realize the precise defect localization, with minimal overlap in detection frames. In addition, the LKSPP module can effectively help the detector mine richer high-level semantics, capture sufficient global information, and take into account local information, even for the most difficult defect.

**Figure 6:**
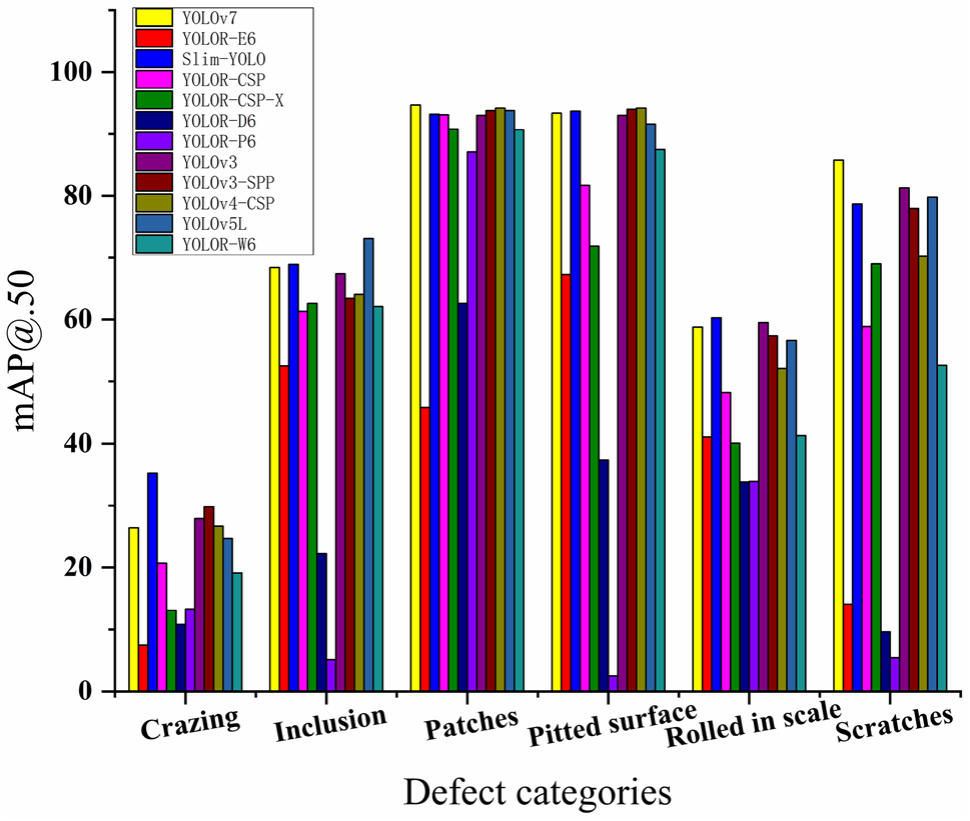
Detection accuracy of the detector for each defect.

Figure 6 shows the accuracies of the detectors for each defect in the test set, with mAP@.50 as the criterion. Slim-YOLO exhibits the highest accuracy in ‘Crazing’ and ‘Rolled in scale’ defect detection, while YOLOR-D6 performs the poorest. YOLOv7 and YOLOv5L perform the best for ‘Scratches’ and ‘Inclusion’, respectively. In the remaining types of defect detection, Slim-YOLO maintains a high level of accuracy. In summary, Slim-YOLO holds an absolute advantage in the defect detection task.

### Ablation study

In this paper, ablation experiments are conducted to demonstrate the significant performance enhancement of the object detector by the proposed module. The specific results are shown in Table 2. With YOLOv7 as the baseline, modules are incrementally added.

**Table 2:** Compare the impact of different proposed modules on the baseline.

MDFFAM	LKSPP	Param. (M)	FLOPs (G)	mAP50 (%)	mAP50:75 (%)	mAP50:95 (%)
Baseline		36.5	103.2	71.2	53.7	37.1
√	×	36.8	103.9	73.0	55.6	38.6
×	√	32.1	99.7	72.5	54.1	37.4
√	√	34.9	103.1	73.4	55.4	38.4

Firstly, in terms of the parameters and computation, adding MDFFAM to YOLOv7 only induces a marginal increase of 0.82% and 0.67%, respectively, more than the original. This indicates that MDFFAM is lightweight enough to disregard the computational overhead it introduces to the detector, while yielding a notable improvement in the detector’s accuracy. In the individual module comparison, YOLOv7 with MDFFAM achieves the highest ${mAP}_{50}$, exhibiting a 1.8% enhancement over the baseline, along with 1.9% and 1.5% improvements in the accuracy metrics ${mAP}_{50:75}$ and ${mAP}_{50:95}$, respectively. Next, testing LKSPP, it is important to note that only the SPPCSPC in YOLOv7 is replaced with LKSPP, while the remainder of the architecture remains unchanged. It is found that the parameters are reduced by 13.7% compared to the baseline. This fully illustrates that the proposed large kernel design principle can maximize the reduction of the parameters and computation. In addition, a series of large kernels in the design improves the effective receptive field of the module and captures more comprehensive features than the paradigm of directly paralleling multiple large kernels. LKSPP demonstrates improvements of 1.3%, 0.4%, and 0.3% over SPPCSPC for ${mAP}_{50}$, ${mAP}_{50:75}$ and ${mAP}_{50:95}$, respectively. Finally, two modules are added to the baseline to achieve the optimal results in three accuracy metrics: ${mAP}_{50}$, ${mAP}_{50:75}$, and ${mAP}_{50:95}$, with an improvement of 2.2%, 1.7%, and 1.3%, respectively. The complexity of the model is further optimized with a 4.6% reduction in the parameters.

Figure 8a illustrates the comparison of classification loss before and after the addition of the module to the baseline model YOLOv7. The incorporation of both modules simultaneously results in a consistent minimization of loss values throughout the entire training process. In particular, with the addition of the modules, the classification performance of YOLOv7 is significantly improved and the loss pattern is smoother. This observation underscores the synergistic effect engendered by the conjoined operation of LKSPP and MDFFAM, attributed to their disparate functional focuses. LKSPP is adept at harnessing rich high-level semantic features owing to its expansive receptive field, while MDFFAM excels in ascertaining precise feature location information. The detector, fortified with the merits of both modules, exhibits a marked enhancement in classification efficacy.

### The importance of MDFFAM

To demonstrate the effectiveness of the proposed MDFFAM in improving the detection performance of the model for small targets, YOLOv7 is used as the baseline and different attention modules are added separately, with results shown in Table 3. The test involves four attention mechanisms: CA, CBAM, SE, and MDFFAM. In terms of the parameters, CBAM, CA, and MDFFAM all operate at the same level, while SE increases the parameters by 3.2% compared to the baseline. Regarding computation load, MDFFAM imposes a relatively small burden, with 14% less computational effort than SE. The difference between MDFFAM and CA, which incurs the least computational overhead, is almost negligible, as MDFFAM is only 0.58% higher compared to CA. Meanwhile, MDFFAM achieves the highest ${mAP}_{50}$ of 73.0, which is 4.7% better than the second-ranked CA, outperforming the baseline by 1.9% and 1.5% in the metrics ${mAP}_{50:75}$ and ${mAP}_{50:95}$, respectively.

**Table 3:** Compare the impact of different attention mechanisms on the baseline.

Models	Param. (M)	FLOPs (G)	mAP50 (%)	mAP50:75 (%)	mAP50:95 (%)
YOLOv7	36.5	103.2	71.2	53.7	37.1
Add CBAM	36.8	106.7	66.1	45.9	30.7
Add SE	37.7	118.4	67.2	48.4	32.9
Add CA	36.7	103.3	68.3	50.7	34.8
Add MDFFAM	36.8	103.9	73.0	55.6	38.6

**Figure 7:**
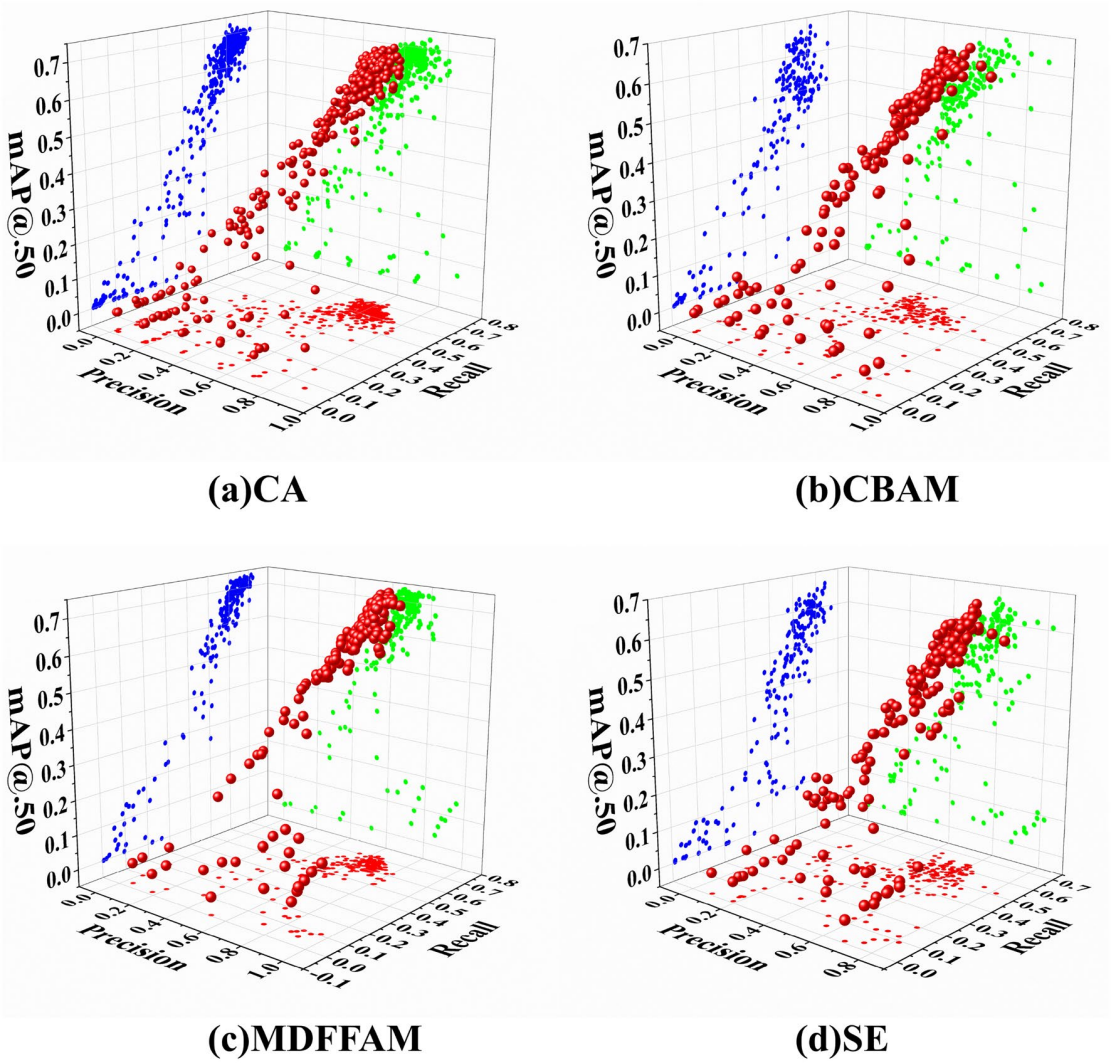
Three-dimensional display of four attention mechanisms.

To better observe the association regulation of Precision, Recall, and ${mAP}_{50}$ for the four attention mechanisms throughout the training phase, a three-dimensional scatter plot is chosen for display, as shown in Fig. 7. At the beginning of the training phase, the results exhibit a scattered distribution. However, as the epoch keeps increasing, the three indicators converge in the same direction, and the scores improve. The figure demonstrates that MDFFAM rapidly enters the convergence state compared with the other three attention mechanisms, with the most minor dispersion fluctuation of the results of MDFFAM in the early training phase. The above experimental results highlight MDFFAM's capacity to facilitate model convergence and maintain stability. From the perspectives of both computational loss and accuracy, MDFFAM exhibits excellent performance.

Except for MDFFAM, the remaining three attention mechanisms all reduce the accuracy of the baseline. This fully illustrates that among the four attention mechanisms, MDFFAM introduces a small computational overhead to the model and also effectively improves detection accuracy. Compared with the other three attention mechanisms, the use of MDFFAM provides greater flexibility to the model.

### The impact of hyperparameter r

To further observe the effect of hyperparameter ‘r’ in the MDFFAM on the model performance, experiments are conducted with YOLOv7 as the baseline. Five sets of experiments are performed to increase the reduction rate ‘r’ from 2 to 32 sequentially to observe the change in performance, and the experimental results are shown in Table 4. The experiments reveal that the maximum number of parameters and computation occurs when the reduction rate is set to the smallest 2. Conversely, the computational burden of the model is the smallest when ‘r’ is set to 32. This indicates that the hyperparameter ‘r’ can flexibly modulate the capacity and computational overhead of the module in the model. Moreover, it is observed that as 'r' increases, the computational overhead diminishes. However, the only goal is not to achieve a lightweight model, accuracy remains of great importance.

**Table 4:** The impact of MDFFAM on the baseline under different settings. Here, r is the reduction rate.

Ratio r	Param. (M)	FLOPs (G)	mAP50 (%)	mAP50:75 (%)	mAP50:95 (%)
2	37.1	106.8	70.8	52.2	36.2
4	36.9	105.2	73.2	56.0	38.4
8	36.8	104.3	71.8	53.0	36.1
16	36.8	103.9	73.0	55.6	38.6
32	36.8	103.7	69.7	51.9	35.7

**Figure 8:**
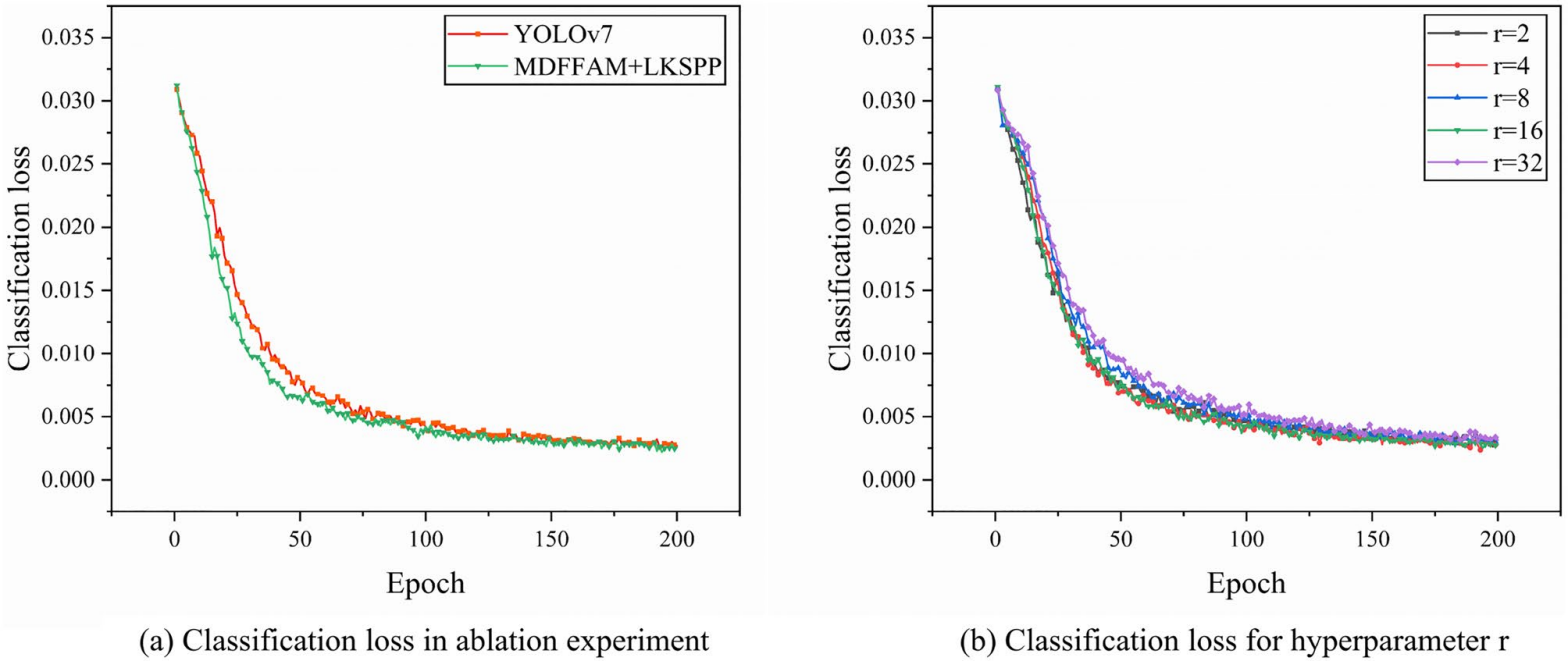
Comparison of classification loss.

Figure 8b illustrates the variations in classification loss of the baseline model throughout the training phase under the influence of different hyperparameters r. A pronounced elevation and frequent oscillations in loss value are observed with r set to 32. Conversely, an assignment of 16 to r yields the most stable and reduced loss value, as evidenced by the smoothest trajectory of the curve. The remaining loss curves exhibit comparable magnitudes and trends, indicating a lesser dependency on the specific value of r within those ranges. Therefore, based on the results, the optimal balance between accuracy and model complexity is obtained when the reduction rate is set to 16, and the reduction rate of 16 is also employed by MDFFAM in the attention mechanism ablation experiment.

## Discussion and conclusion

Much research has been conducted on object detection. CNNs^47,48^ are employed to extract object features for the detection task. The enhancement of network depth^49^ is a chosen strategy to improve the detection accuracy. The relation network^50^ can boost detectors’ effective integration of the extracted feature information. YOLOv7, as a state-of-the-art single-stage detection algorithm, is capable of quick and comprehensive detection tasks. Under unfavorable conditions such as insufficient light and shadows, GAFF^51^ can fuse the visible and thermal features of the target to further weaken external interference. CPFM^52^ mines the precise features across different modes and fuses them in a complementary way to enhance the robustness of the detection.

This paper proposes two new components: the MDFFAM and the LKSPPF. MDFFAM can make full use of spatial location information to assist the model in the accurate identification of the detection focus while ensuring the establishment of stable long-range dependencies. On the other hand, LKSPP not only flexibly handles inputs of varying scales and sizes but also obtains richer and more advanced semantic features, which is mainly attributed to the effective receptive field expansion enabled by large kernels. Furthermore, the serial connection of several large kernels in LKSPP further suppresses the redundancy in the computational burden associated with large kernels. The obtained effective receptive field is larger for series than for parallel. Experimental results empirically validate that the detector assembled with MDFFAM and LKSPP as the core achieves highly competitive performance in small object detection tasks. Additionally, when testing the MDFFAM and LKSPP modules in isolation, both demonstrate a decent performance in their respective comparative experiments. This shows that the incorporation of MDFFAM or LKSPP into the baseline independently induces obvious improvement in model performance.

The complexity of mechanical structures can result in surface defects not readily discernible under normal lighting conditions or partially visible in shadow. Therefore, there is a great interest in future research regarding data enhancement tools based on the fusion of thermal and visible imaging features. The next work will focus on an effective combination of the feature fusion methods from the two different imaging with large kernel and attention mechanisms. The approach aims to enhance the robustness of the detector and its accuracy.

## Data Availability

The paper contains all research data.

## References

[1] Ge, Z., Liu, S., & Wang, F., *et al.* YOLOX: Exceeding YOLO series in 2021 [J]. 10.48550/arXiv.2107.08430 (2021).

[2] Bochkovskiy, A., Wang, C. Y., & Liao, H. Y. M. YOLOv4: Optimal speed and accuracy of object detection [J]. 10.48550/arXiv.2004.10934 (2020).

[3] Redmon, J., Divvala, S., Girshick, R., *et al.* You only look once: Unified, real-time object detection [C]. 10.1109/CVPR.2016.91 (pp.779–788) (2016).

[4] Redmon, J., & Farhadi, A. YOLO9000: Better, faster, stronger [C]. pp. 6517–6525. 10.1109/CVPR.2017.690 (2017).

[5] Redmon, J., & Farhadi, A. YOLOv3: An incremental improvement [J]. 10.48550/arXiv.1804.02767 (2018).

[6] Wang, C. Y., Bochkovskiy, A., Liao, H. Y. M. Scaled-YOLOv4: Scaling cross stage partial network [J]. 10.48550/arXiv.2011.08036 (2020).

[7] Tian, Z., Shen, C., Chen, H., *et al.* FCOS: Fully convolutional one-stage object detection [C]. pp. 9626–9635. 10.1109/ICCV.2019.00972 (2019).

[8] Tian, Z., Shen, C., Chen, H., *et al.* FCOS: A simple and strong anchor-free object detector [J]. pp. 1922–1933. 10.1109/TPAMI.2020.3032166 (2022). 10.1109/TPAMI.2020.3032166 33074804

[9] Wang, C. Y., Bochkovskiy, A., & Liao, H. Y. M. YOLOv7: Trainable bag-of-freebies sets new state-of-the-art for real-time object detectors [J]. 10.48550/arXiv.2207.02696 (2022).

[10] Lin T-Y, Dollár P, Girshick R, He K, Hariharan B, Belongie S (2017). Feature pyramid networks for object detection..

[11] Liu, S., Huang, D., & Wang, Y. Learning spatial fusion for single-shot object detection [J]. 10.48550/arXiv.1911.09516 (2019).

[12] Hu, H., Gu, J., & Zhang, Z., *et al.* Relation networks for object detection [C]. pp. 3588–3597. 10.48550/arXiv.1711.11575 (2018).

[13] Jiang M, Li G, Xie L (2017). Adaptive classifier for steel strip surface defects [J]. J. Phys. Conf. Ser..

[14] Park Y, Kweon IS (2016). Ambiguous surface defect image classification of AMOLED displays in smartphones. IEEE Trans. Ind. Inf..

[15] Chu M (2017). Multi-class classification for steel surface defects based on machine learning with quantile hyper-spheres. Chemom. Intell. Lab. Syst..

[16] Liu K, Wang H, Chen H, Qu E, Tian Y, Sun H (2017). Steel surface defect detection using a new Haar–Weibull-variance model in unsupervised manner. IEEE Trans. Instrum. Meas..

[17] Chu M, Gong R, Gao S (2017). Steel surface defects recognition based on multi-type statistical features and enhanced twin support vector machine—ScienceDirect [J]. Chemom. Intell. Lab. Syst..

[18] Dosovitskiy, A., Beyer, L., Kolesnikov, A., *et al.* An image is worth 16 × 16 words: Transformers for image recognition at scale [J]. 10.48550/arXiv.2010.11929 (2021).

[19] Vaswani, A., Shazeer, N., Parmar, N., *et al.* Attention is all you need [J]. 10.48550/arXiv.1706.03762 (2017).

[20] Cordonnier, J. B., Loukas, A., & Jaggi, M. On the relationship between self-attention and convolutional layers [J]. 10.48550/arXiv.1911.03584 (2019).

[21] Paul, S., & Chen, P. Y. Vision transformers are robust learners [J]. 10.48550/arXiv.2105.07581 (2021).

[22] Raghu, M., Unterthiner, T., & Kornblith, S., *et al.* Do vision transformers see like convolutional neural networks? [C]. 10.48550/arXiv.2108.08810 (2021).

[23] Xie, E., Wang, W., Yu, Z., *et al.* SegFormer: Simple and efficient design for semantic segmentation with transformers [J]. 10.48550/arXiv.2105.15203 (2021).

[24] He, K., Zhang, X., & Ren, S., *et al.* Deep residual learning for image recognition [J]. pp. 770–778. 10.1109/CVPR.2016.90 (2016).

[25] Krizhevsky A, Sutskever I, Hinton G (2012). ImageNet classification with deep convolutional neural networks [J]. Commun. ACM.

[26] Ding, X., Zhang, X., Zhou, Y., *et al.* Scaling up your kernels to 31x31: Revisiting large kernel design in CNNs [J]. 10.48550/arXiv.2203.06717 (2022).

[27] Liu, Z., Mao, H., Wu, C.Y., *et al.* A ConvNet for the 2020s [J]. 10.48550/arXiv.2201.03545 (2022).

[28] Liu, Z., Lin, Y., Cao, Y.,* et al.* Swin transformer: Hierarchical vision transformer using shifted windows [J]. 10.48550/arXiv.2103.14030 (2021).

[29] Han, Q., Fan, Z., Dai, Q., *et al.* Demystifying local vision transformer: sparse connectivity, weight sharing, and dynamic weight [J]. 10.48550/arXiv.2106.04263 (2021).

[30] Trockman, A., & Zico Kolter, J. Patches are all you need? [J]. 10.48550/arXiv.2201.09792 (2022).

[31] Hu, J., Shen, L., & Sun, G. Squeeze-and-excitation networks [C]. pp. 7132–7141. 10.1109/CVPR.2018.00745 (2018).

[32] Woo, S., Park, J., Lee, J. Y., *et al.* CBAM: Convolutional block attention module [J]. pp. 3–19. 10.1007/978-3-030-01234-2_1 (ECCV, 2018).

[33] Hou, Q., Zhou, D., & Feng, J. Coordinate attention for efficient mobile network design [J]. 10.48550/arXiv.2103.02907 (2021).

[34] Zhao, H., Jia, J., & Koltun, V. Exploring self-attention for image recognition [C]. pp. 10073–10082. 10.1109/CVPR42600.2020.01009 (2020).

[35] Srinivas, A., Lin, T. Y., Parmar, N., *et al.* Bottleneck transformers for visual recognition [J]. pp. 16514–16524. 10.1109/CVPR46437.2021.01625 (2021).

[36] Bello I, Zoph B, Vaswani A (2019). Attention augmented convolutional. Networks..

[37] Gao, P., Lu, J., Li, H., *et al.* Container: Context aggregation network [J]. 10.48550/arXiv.2106.01401 (2021).

[38] Liu, J. J., Hou, Q., Cheng, M. M., *et al.* Improving convolutional networks with self-calibrated convolutions [C]. pp. 10093–10102. 10.1109/CVPR42600.2020.01011 (2020).

[39] Wang X, Girshick R, Gupta A (2018). Non-local. Neural Netw..

[40] Gao, Z., Xie, J., Wang, Q., *et al.* Global second-order pooling convolutional networks [C]. pp. 3019–3028. 10.1109/CVPR.2019.00314 (2019).

[41] He, K., Zhang, X., Ren, S. & Sun, J. Spatial Pyramid Pooling in Deep Convolutional Networks for Visual Recognition. 1904–1916. 10.1109/TPAMI.2015.2389824 (2015). 10.1109/TPAMI.2015.2389824 26353135

[42] Glenn J. YOLOv5 release v6.1. https://github.com/ultralytics/yolov5/releases/tag/v6.1 (2022).

[43] Bodla, N., Singh, B., Chellappa, R., *et al.* Soft-NMS—Improving Object Detection with One Line of Code[C]. pp. 5562–5570. 10.1109/ICCV.2017.593 (2017).

[44] Ning, C., Zhou, H., Song, Y., *et al.* Inception single shot multibox detector for object detection [C]. pp. 549–554. 10.1109/ICMEW.2017.8026312 (2017).

[45] He, Y., Song, K., Meng, Q., & Yan, Y. *An end-to-end steel surface defect detection approach via fusing multiple hierarchical features*. pp. 1493–1504. 10.1109/TIM.2019.2915404 (2020).

[46] Wang, C. Y., Yeh, I. H., Liao, H. Y. M. *You Only Learn One Representation: Unified Network for Multiple Tasks[J].*10.48550/arXiv.2105.04206 (2021).

[47] Yi L, Li G, Jiang M (2016). An end-to-end steel strip surface defects recognition system based on convolutional neural networks [J]. Steel Res. Int..

[48] Zhou S, Chen Y, Zhang D (2017). Classification of surface defects on steel sheet using convolutional neural networks [J]. Mater. Technol..

[49] Natarajan, V., Hung, T. -Y., Vaikundam, S., & Chia, L. -T. Convolutional networks for voting-based anomaly classification in metal surface inspection. pp. 986-991. 10.1109/ICIT.2017.7915495 (2017).

[50] Hu, H., Gu, J., Zhang, Z., Dai, J., & Wei, Y. Relation networks for object detection (pp. 3588–3597). 10.1109/CVPR.2018.00378 (2018).

[51] Zhang, H., Fromont, E., Lefevre, S., & Avignon, B. Guided attentive feature fusion for multispectral pedestrian detection. pp. 72–80. 10.1109/WACV48630.2021.00012 (2021).

[52] Li, M., Shao, Z., Shi, Z., *et al.* Deep visible and thermal image fusion with cross-modality feature selection for pedestrian detection [C] (2020).

